# The mechanisms and drug therapies of colorectal cancer and epigenetics: bibliometrics and visualized analysis

**DOI:** 10.3389/fphar.2024.1466156

**Published:** 2024-08-29

**Authors:** Siyu Tian, Min Chen

**Affiliations:** ^1^ School of Clinical Medicine, Chengdu University of TCM, Chengdu, China; ^2^ Hospital of Chengdu University of Traditional Chinese Medicine, Chengdu, China

**Keywords:** colorectal cancer, epigenetics, mechanisms, drug therapies, bibliometrics

## Abstract

**Background:**

Numerous studies have demonstrated a link between epigenetics and CRC. However, there has been no systematic analysis or visualization of relevant publications using bibliometrics.

**Methods:**

839 publications obtained from the Web of Science Core (WoSCC) were systematically analyzed using CiteSpace and VOSviewer software.

**Results:**

The results show that the countries, institutions, and authors with the most published articles are the United States, Harvard University, and Ogino and Shuji, respectively. SEPT9 is a blood test for the early detection of colorectal cancer. Vitamin D and gut microbiota mediate colorectal cancer and epigenetics, and probiotics may reduce colorectal cancer-related symptoms. We summarize the specific epigenetic mechanisms of CRC and the current existence and potential epigenetic drugs associated with these mechanisms. It is closely integrated with clinical practice, and the possible research directions and challenges in the future are proposed.

**Conclusion:**

This study reviews the current research trends and hotspots in CRC and epigenetics, which can promote the development of this field and provide references for researchers in this field.

## 1 Introduction

Colorectal cancer (CRC) remains the leading cause of cancer-related death worldwide. CRC is the third most common cancer worldwide, with a mortality rate of up to 35% in the United States, 45% in Europe, and 47.8% globally (Bray et al., 2018; ECIS, 2019; Siegel et al., 2019). Surgery has become an important treatment for CRC, however, surgical trauma and follow-up care are inconvenient for patients (Kuipers et al., 2015). Therefore, a clinical incision is needed to find an effective treatment for patients with CRC. Epigenetics mediate the development of CRC by altering the expression of heritable genes (Kawakami et al., 2015). Relevant studies have clarified the link between certain CRC-specific genes and epigenetic changes (Dienstmann et al., 2017). For example, microsatellite instability (MSI), in which promoter hypermethylation causes epigenetic changes in genes, is a marker for CRC molecular subpopulations (Herman et al., 1998). Hypomethylation in the human body is also an important factor that leads to chromosome instability in CRC (Suter et al., 2004). In addition, microRNAs (miRNAs) block protein expression in almost all CRC stages and affect many cancer-related pathways (Strubberg and Madison, 2017). For example, miR-143 blocks cell growth through direct targeting and has been found to be frequently downregulated in gene expression of CRC (Chen et al., 2009). Therefore, the study of CRC and epigenetics has deepened our understanding of the pathophysiological mechanisms of CRC, while providing new ideas and directions for the search for biomarkers and therapeutic targets for CRC. Researchers are interested in this area, and an increasing number of specialized studies are emerging. However, The relationship between CRC and epigenetics and the epigenetic drugs associated with CRC have not been systematically bibliometrically and visually analyzed. In-depth bibliometric research of countries, institutions, journals, authors, citations, and keywords for publications relevant to the field is necessary. Bibliometric analysis uses mathematical and statistical methods to quantitatively analyze research priorities and hotspots within a research field and to assess the scientific productivity of countries, institutions, and researchers (Jiang et al., 2023). Therefore, it describes the current research focus, hotspots, and future research development. This study provides an in-depth review of the current status of CRC and epigenetic research between 2011 and 2023, filling a gap in the bibliometric analysis of the literature in this field. CiteSpace and VOSviewer were used for visual analysis of the literature.

## 2 Methods

### 2.1 Data source and search strategy

**FIGURE 1:**
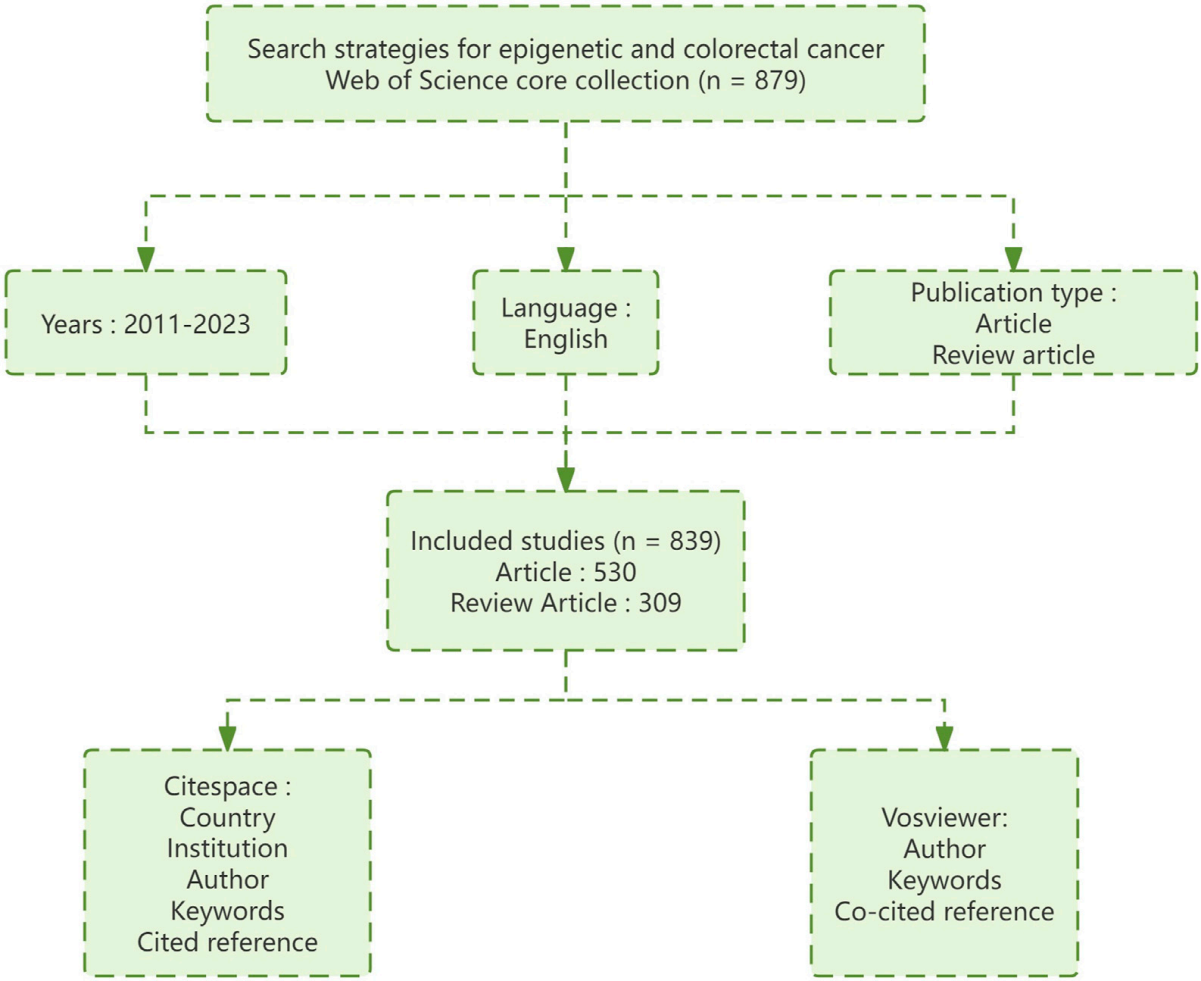
Document retrieval flow chart.

We obtained the literature we needed from the Web of Science Core Collection (WOSCC), which is limited to “English” papers published between 1 January 2011, and 31 December 2023. The Article type is limited to “Article” and “Review.” We searched for topics and free words related to CRC and epigenetics. Finally, the data are exported in plain text format with “full records and citations.” We used the same method to retrieve subject words and free words of epigenetic drugs for CRC. A literature search was conducted independently by two researchers on 12 January 2024. Figure 1 shows the literature search process in this field.

### 2.2 Software for bibliometric analysis and visualization analysis

Microsoft Office Excel 2016, CiteSpace.5.8.R5, and Vosviewer1.6.19 were used in this study. Microsoft Office Excel 2016 was used to create statistics on the annual publication trends, data sorting, and tables. CiteSpace 5.8.R5 was used to analyze countries, institutions, authors, highly cited references, keyword frequency and centrality, and keyword bursts. Authors, journals, and co-citations were analyzed using Vosviewer 1.6.19. In this study, the specific parameters of CiteSpace were set and the results were explained (Tian et al., 2023). The time frame of The study period was from 2011 to 2023. The author, institution, country, and keywords corresponded to different node types.

## 3 Results

### 3.1 Publishing trend analysis

**FIGURE 2:**
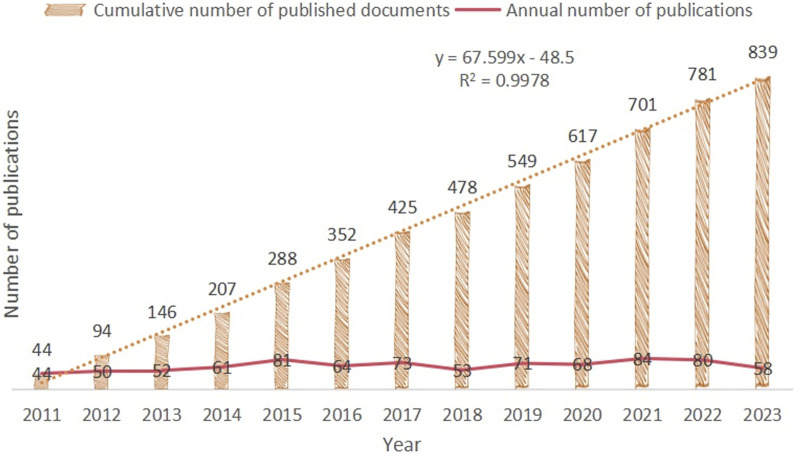
Published Trend Maps on CRC and epigenetics.

This study included 839 relevant publications on CRC and epigenetics. From 2011 to 2023, although the number of publications varied from year to year, the overall trend in this field of research was upward, with a steady increase in the cumulative number of publications, as shown in Figure 2. We used an exponential growth function to assess the correlation between the cumulative number of publications per year and year. There was a strong correlation between the number of publications and the publication year (R^2^ = 0.9978). Using the exponential function, we can calculate that the cumulative number of publications in 2024 may be 898. This strong association indicates that CRC and epigenetics are receiving increasing academic attention. Thus, the study of CRC and epigenetics is attracting increasing interest from researchers. Similarly, Supplementary Figure S1 showed that the annual cumulative number of articles on epigenetic drugs for CRC has also been increasing (R^2^ = 0.987), which has received continuous attention from the scientific community.

### 3.2 Publications of countries/regions, institutions, and authors

We investigated the number of publications on CRC and epigenetics-related research, and the networks of collaboration between countries, institutions, and authors (Table 1). The larger the node in the diagram, the greater the number of posts. The purple outer circle indicates that the centrality value of the medium was higher than 0.1.

**TABLE 1:** Countries/regions, institutions, and authors ranked by publications and centrality.

Item	Rank	Name	Publications	Name	Centrality
Countries/Regions	1	UNITED STATES	246 (30.41%)	UNITED STATE	0.39
	2	PEOPLES R CHINA	205 (25.34%)	GERMANY	0.25
	3	ITALY	71 (8.78%)	ENGLAND	0.19
	4	JAPAN	55 (6.80%)	PEOPLES R CHINA	0.16
	5	SPAIN	48 (5.93%)	SPAIN	0.16
	6	GERMANY	44 (5.44%)	SWEDEN	0.09
	7	ENGLAND	41 (5.07%)	ITALY	0.07
	8	INDIA	41 (5.07%)	JAPAN	0.07
	9	AUSTRALIA	30 (3.71%)	INDIA	0.07
	10	IRAN	28 (3.46%)	AUSTRALIA	0.07
Institutions	1	Harvard University	29 (16.96%)	University of Texas System	0.13
	2	Harvard Medical School	20 (11.70%)	Brigham and Women’s Hospital	0.12
	3	Harvard T.H. Chan School of Public Health	19 (11.11%)	Albert Einstein College of Medicine	0.07
	4	Johns Hopkins University	19 (11.11%)	Ruprecht Karls University Heidelberg	0.07
	5	Brigham and Women’s Hospital	18 (10.53%)	Helmholtz Association	0.06
	6	CIBER—Centro de Investigacion Biomedica en Red	15 (8.77%)	German Cancer Research Center (DKFZ)	0.06
	7	Dana-Farber Cancer Institute	15 (8.77%)	Royal College of Surgeons—Ireland	0.06
	8	Johns Hopkins Medicine	14 (8.19%)	Zhejiang University	0.05
	9	Zhejiang University	11 (6.43%)	UTMD Anderson Cancer Center	0.05
	10	Helmholtz Association	11 (6.43%)	Johns Hopkins University	0.04
Authors	1	Ogino, Shuji	9 (27.27%)	Ogino, Shuji	0.01
	2	Coppede, Fabio	6 (18.18%)	Alwers, Elizabeth	0.01
	3	Nishihara, Reiko	6 (18.18%)	Akimoto, Naohiko	0.01
	4	Ahuja, Nita	6 (18.18%)	Amitay, Efrat L	0.01
	5	Goel, Ajay	6 (18.18%)	Coppede, Fabio	0.00

#### 3.2.1 Analysis of national publications and collaborations

**FIGURE 3:**
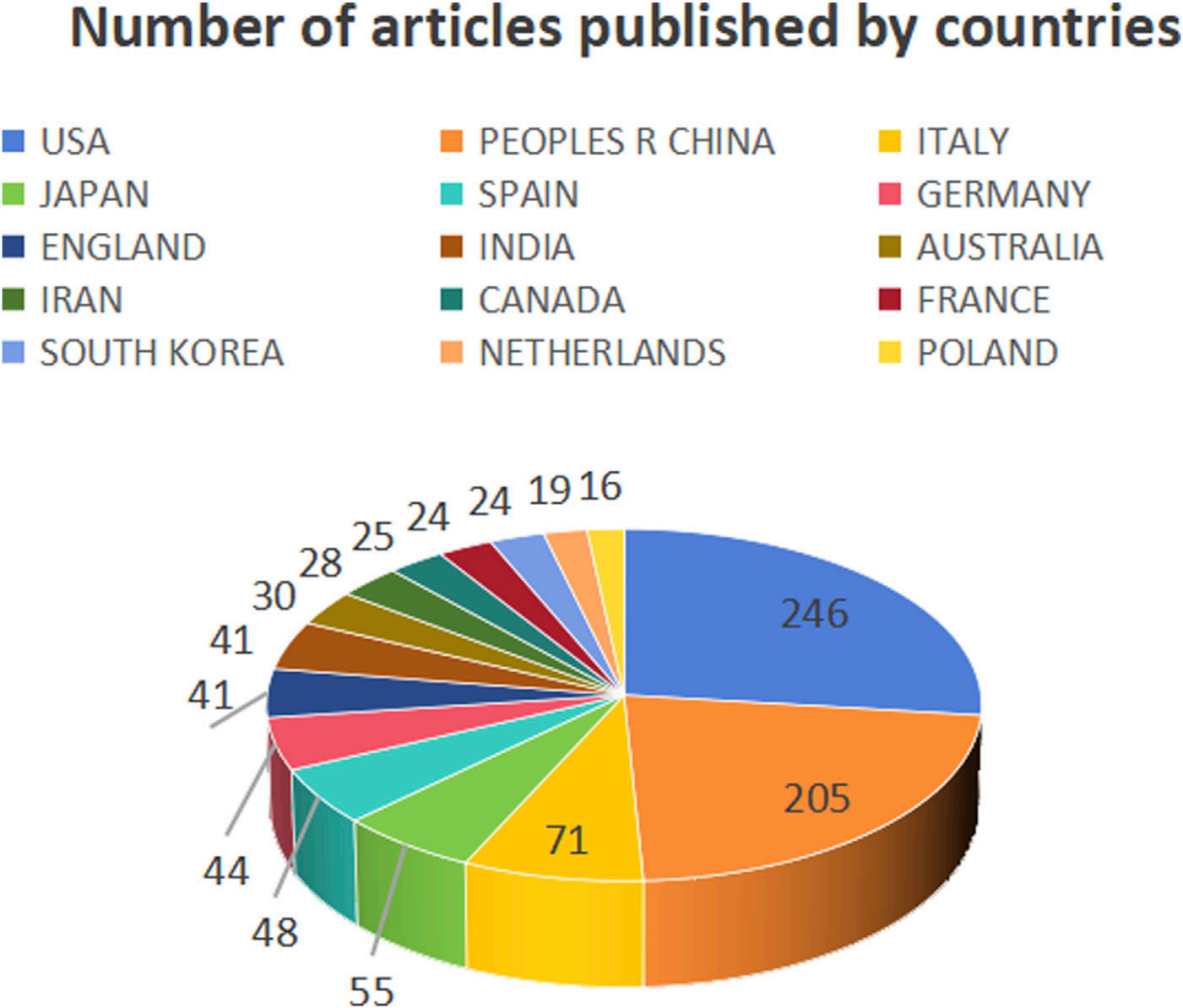
Number of publications by countries.

**FIGURE 4:**
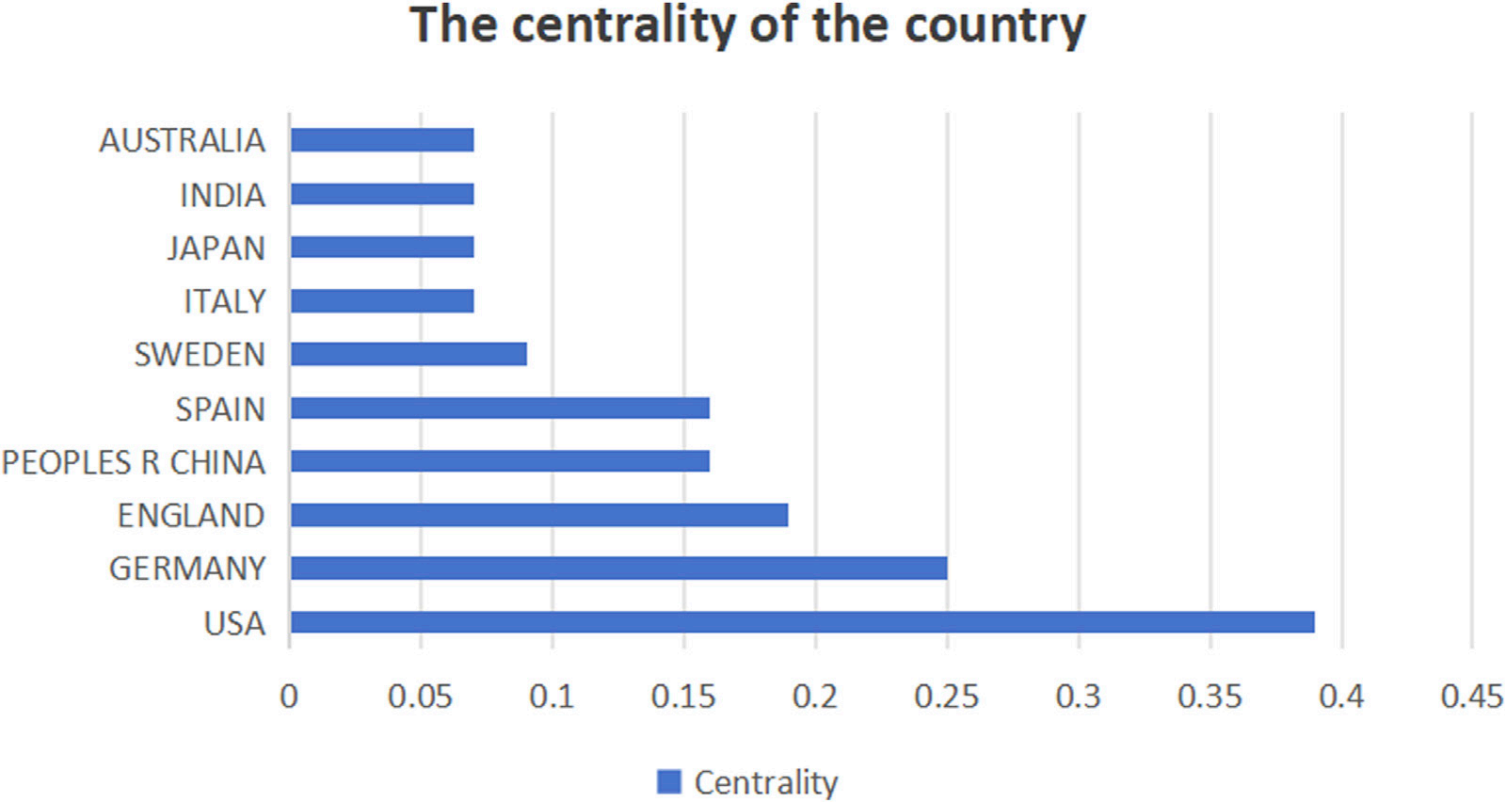
Intermediary centrality of countries.

**FIGURE 5:**
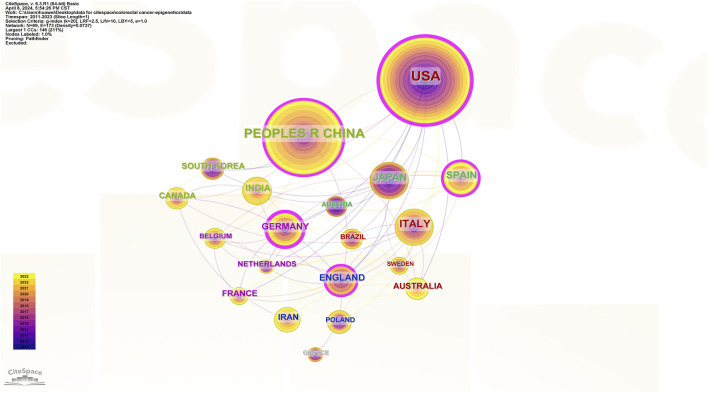
Collaborative networks of countries.

This study analyzed the number of publications in different countries (Figure 3), centrality (Figure 4), and synergy networks between CRC and epigenetic-related research (Figure 5). The results of the study in Figures 3, 4 show that the United States (246 publications, 30.41%), PEOPLES R CHINA (205 publications, 25.34%), ITALY (71 publications, 8.78%), JAPAN (55 publications, 6.80%), and SPAIN (48 publications, 5.93%) had the highest number of published papers. In addition, the UNITED STATES (0.39), GERMANY (0.25), ENGLAND (0.19), PEOPLES R CHINA (0.16), and SPAIN (0.16) are the top five countries with the strongest country centralities in the field, representing their close cooperation with other countries. The number of publications and country-specific information on centrality are presented in Table 1.

#### 3.2.2 Analysis of institutional publications and collaborations

**FIGURE 6:**
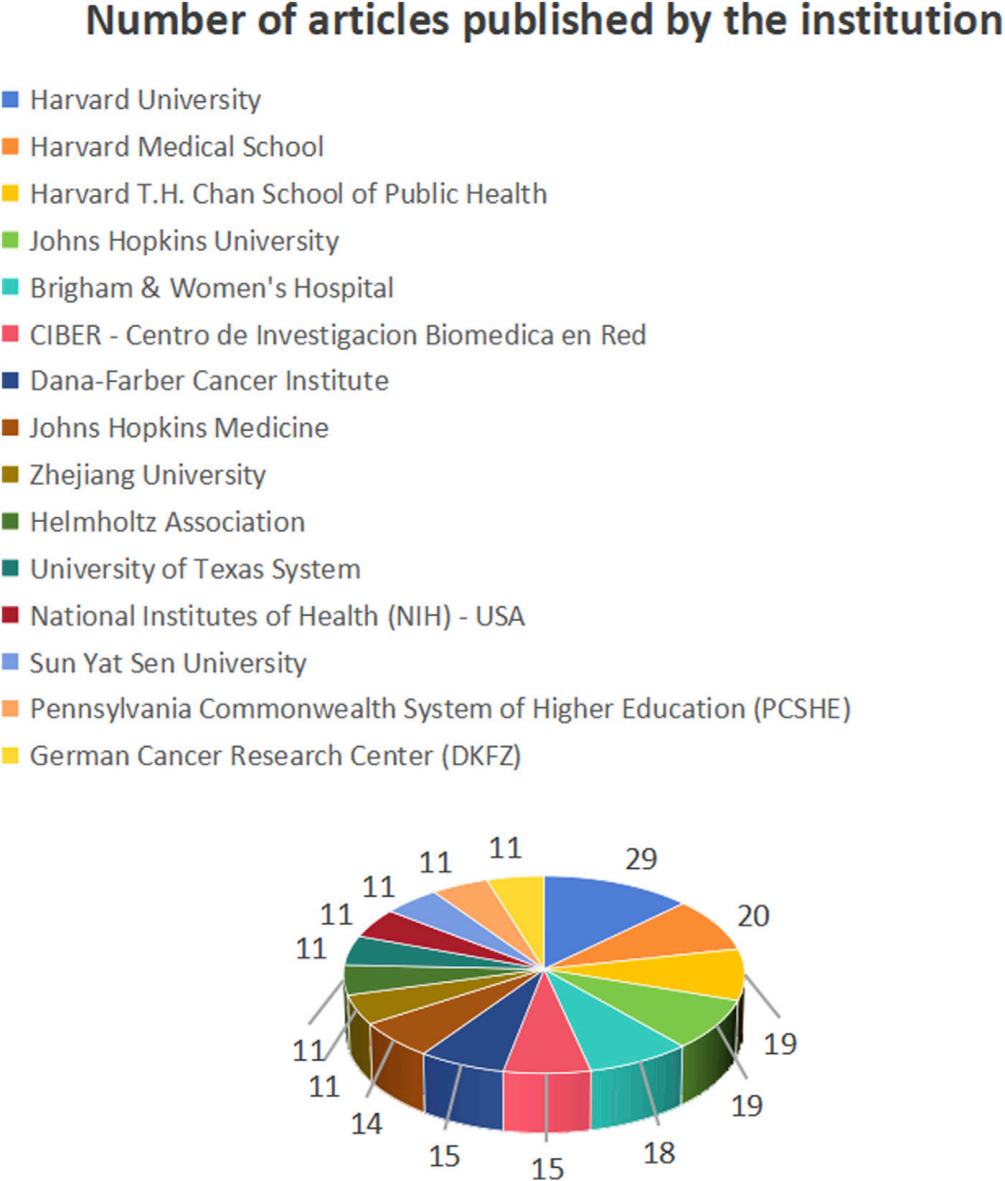
Number of publications by institution.

**FIGURE 7:**
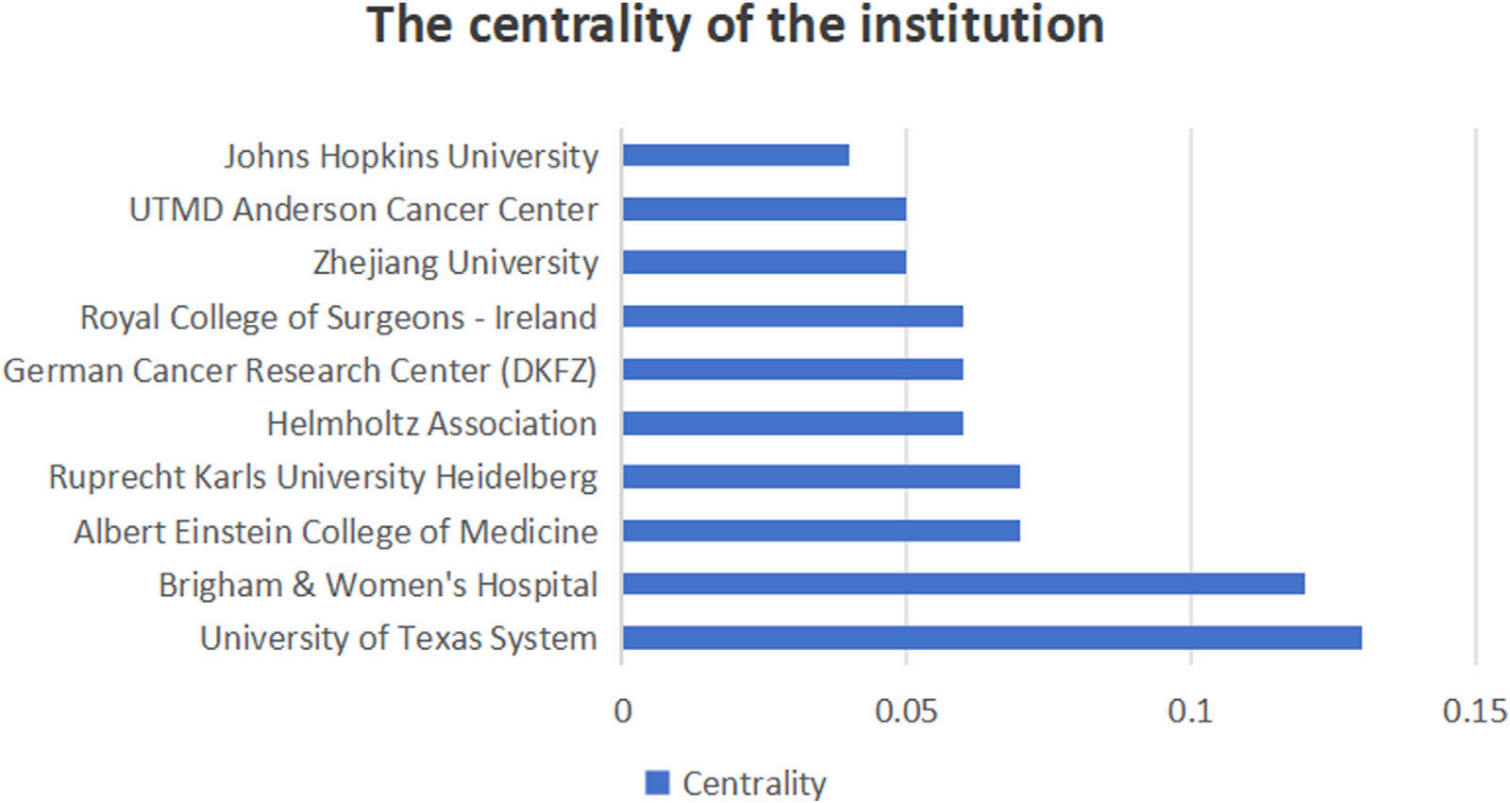
Intermediary centrality of institutions.

**FIGURE 8:**
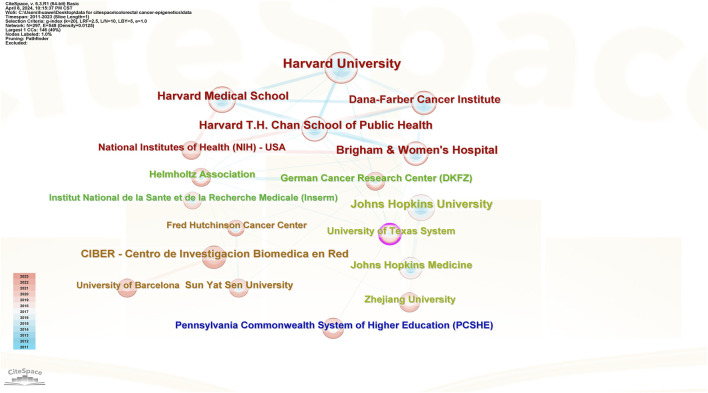
Collaborative networks of institutions.

Figure 6 shows the number of publications from institutions, Figure 7 shows the centrality between institutions, and Figure 8 illustrates the network of collaboration between institutions. As shown in Figure 6, Harvard University (29 publications, 16.96%), Harvard Medical School (20 publications, 11.70%), Harvard T.H. Chan School of Public Health (19 publications, 11.11%), Johns Hopkins University (19 publications, 11.11%), and Brigham and Women’s Hospital (18 publications, 10.53%). The world’s top cutting-edge research institutions have contributed the largest amount of literature in this field, indicating that this research field is at the forefront of world research and has attracted wide attention from scholars around the world. In addition, the University of Texas System (0.13) and Brigham and Women’s Hospital (0.12), with institutional center values greater than 0.1, are the most closely aligned institutions in their field. Specific information regarding the number of publications and institutions is presented in Table 1.

#### 3.2.3 Analysis of publications and cooperation among authors

**FIGURE 9:**
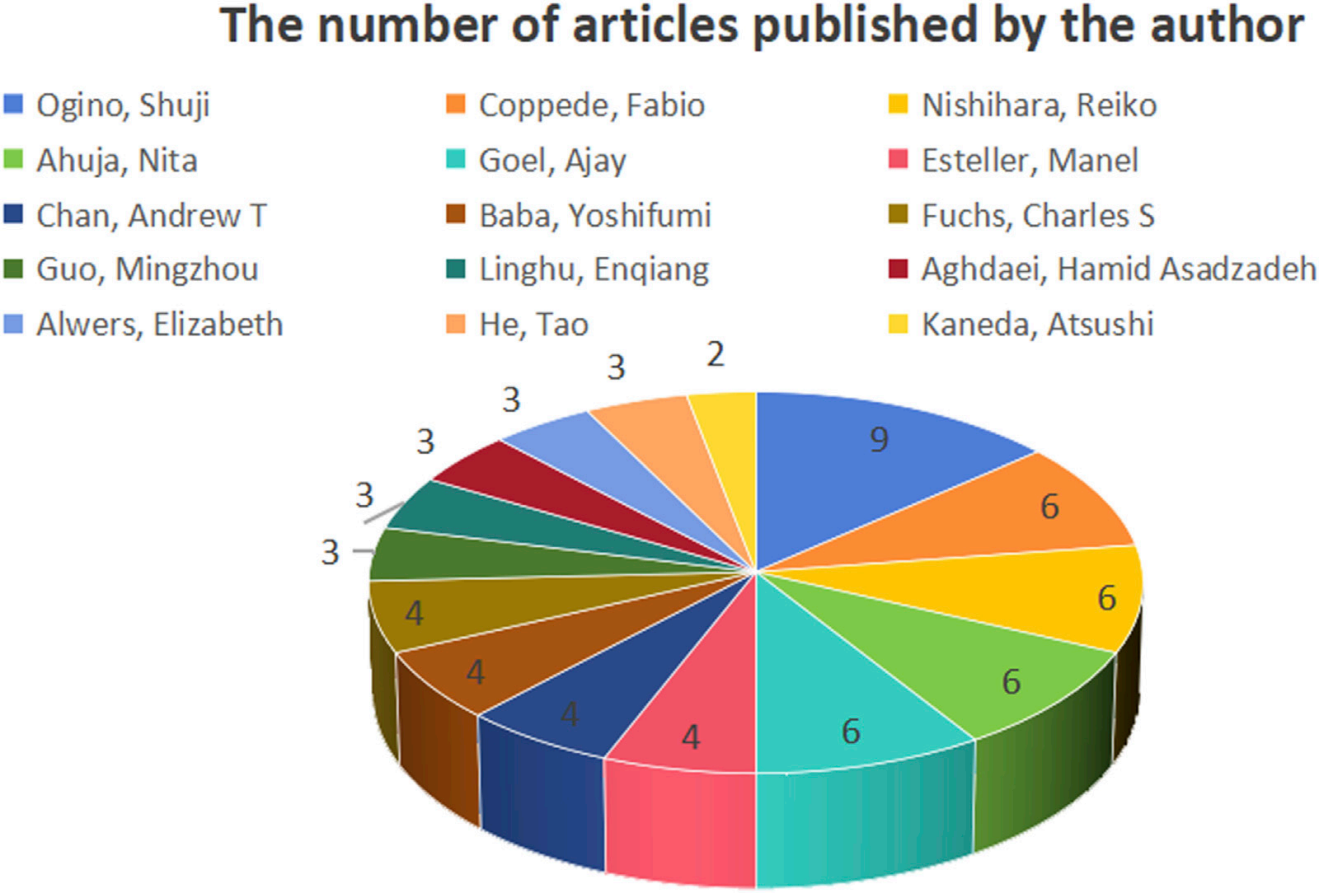
Number of publications by authors.

**FIGURE 10:**
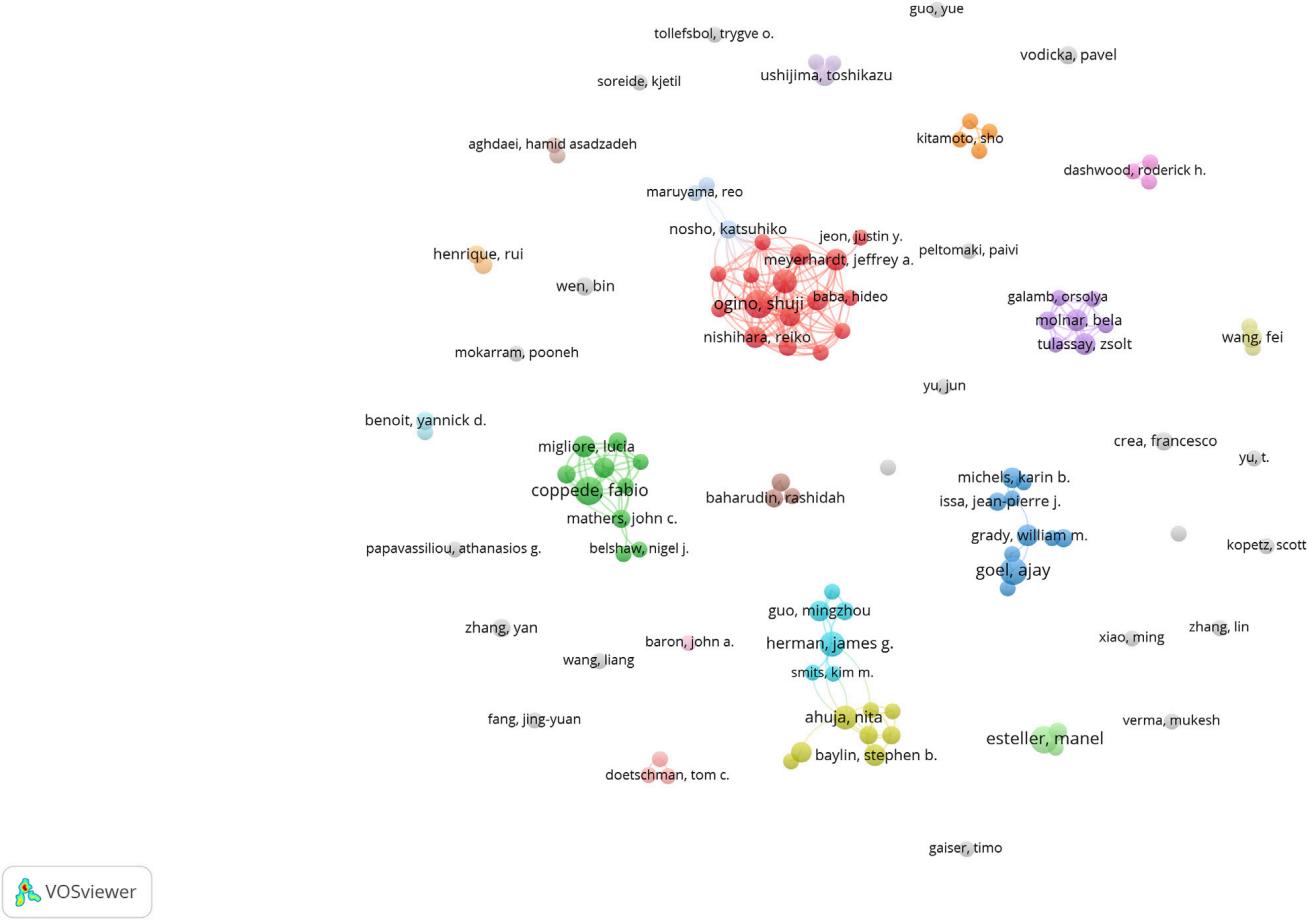
Collaborative networks of authors.

Figure 9 shows the number of publications by the authors, while Figure 10 illustrates the network of collaboration between authors. As shown in Figure 9, Ogino, Shuji (9 publications, 27.27%), Coppede Fabio (6 publications, 18.18%), Nishihara, Reiko (6 publications, 18.18%), Ahuja, Nita (6 publications, 18.18%), and Goel, Ajay (6 publications, 18.18%) were the top five authors with the most published articles in this field. Cooperation among authors was not high, except for Ogino, Shuji (0.1), Alwers, Elizabeth (0.1), Akimoto, Naohiko (0.1), Amitay, and Efrat L (0.1), whose centrality was 0.1, and other authors whose centrality was 0. Detailed information is provided in Table 1. Governments and institutions should promote cooperation among authors and increase a large amount of financial support to promote the development of this research field.

### 3.3 Research hot spots and trend analysis

#### 3.3.1 Analysis of highly co-cited references

**FIGURE 11:**
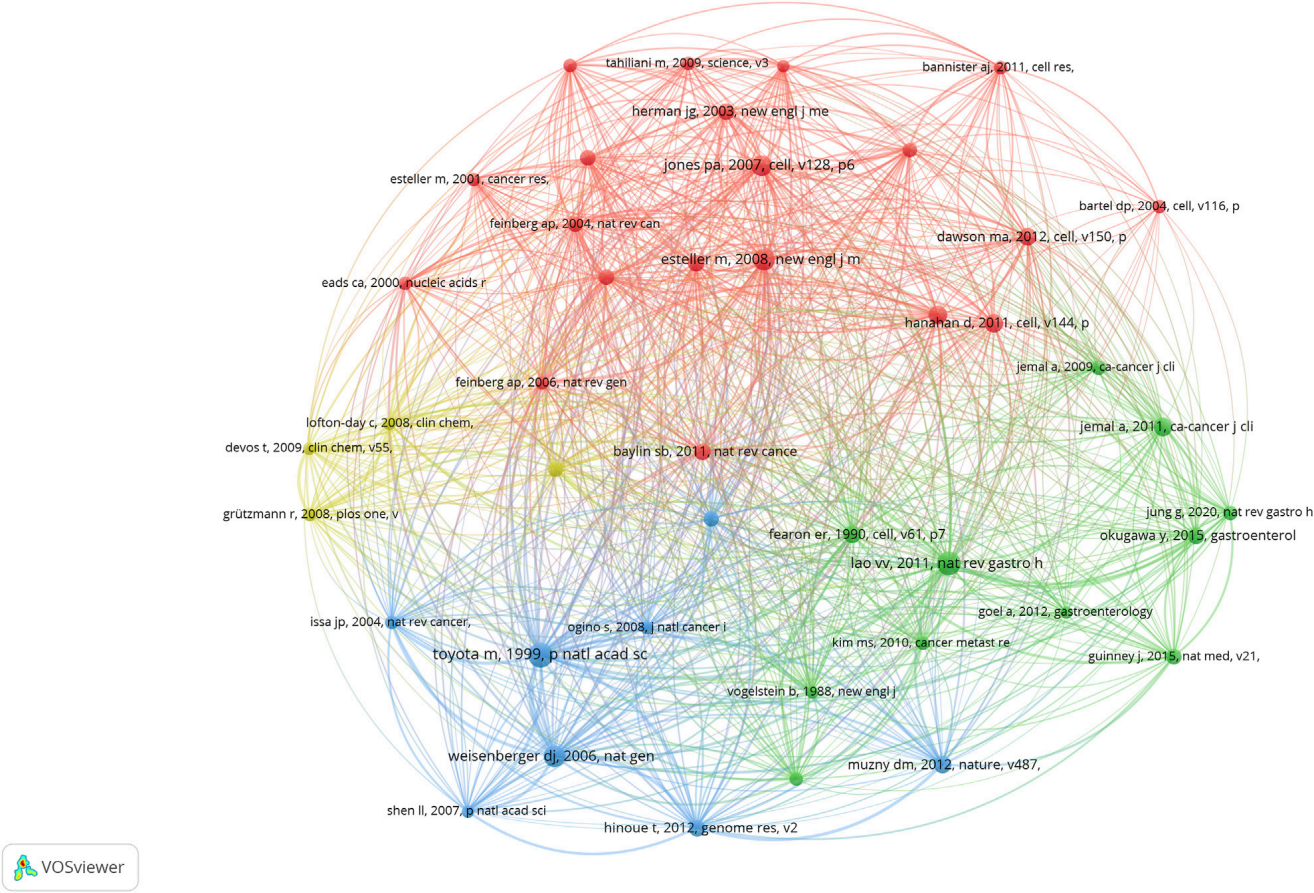
Cluster mapping of highly co-cited literature.

We use VOSviewer to study the co-cited references in this field, and the total number of articles was 52,024 (Table 2). The number of references generated in the analysis was reduced to 48, when the minimum reference was set to 26. Figure 11 shows this diagram. The highly co-cited references in the network map can be divided into four groups, each represented by a different color: red, green, blue, and yellow. The literature in the red cluster is mainly a review of CRC and epigenetics, mainly showing how epigenetics is involved in the latest progress in the early stage of cancer and discussing the impact of epigenetics on cancer control (Esteller, 2008; Jones and Baylin, 2007; Hanahan and Weinberg, 2011). The green cluster of literature focuses on the mechanism between CRC and epigenetics, including the abnormal methylation of CRC genes and the discovery of CpG island methylation phenotypes (CIMP) (Kim et al., 2010; Weisenberger et al., 2006). Emerging biomarkers have also been identified in CRC epigenetics (Okugawa et al., 2015; Goel and Boland, 2012). The literature in the blue cluster is dominated by genomic analyses of CRC, in which three-quarters of the genes are accompanied by high MSI and hypermethylation (Hinoue et al., 2012; Kawakami et al., 2015). The yellow cluster literature mainly focuses on DNA methylation analysis of SEPT9 in plasma, which is suitable for epigenetic detection of CRC and mediates the early detection of CRC (Grützmann et al., 2008; deVos et al., 2009; Church et al., 2014). Among the top ten co-cited literature, we found that basic research on epigenetics and CRC mainly focused on related mechanisms, and the top three cited studies were mainly related to CIMP (Toyota et al., 1999; Lao and Grady, 2011), which is the basis of MSI. CRC is closely associated with BRAF mutations (Weisenberger et al., 2006).

**TABLE 2:** Top 10 highly co-cited references.

Item	Rank	Title	Journal	Citation
Co-cited references	1	CpG island methylator phenotype in colorectal cancer	PNAS	98
	2	Epigenetics and colorectal cancer	Nature Reviews Gastroenterology and Hepatology	88
	3	CpG island methylator phenotype underlies sporadic MSI and is tightly associated with BRAF mutation in colorectal cancer	Nature Genetics	76
	4	Epigenetics in cancer	The New England Journal of Medicine	73
	5	The epigenomics of cancer	Cell	72
	6	Hallmarks of cancer: the next-generation	Cell	58
	7	Epigenetics in cancer. Carcinogenesis	Carcinogenesis	57
	8	A genetic model for colorectal tumorigenesis	Cell	52
	9	Comprehensive molecular characterization of human colon and rectal cancer	Nature	51
	10	Cancer epigenetics: from mechanism to therapy	Cell	50

#### 3.3.2 Analysis of highly cited references

The highly cited literature embodies both academic and professional significance. We analyzed the top 10 citations in the field of CRC and epigenetic research. The two most cited articles were published in the journal NAT REV GASTRO HEPAT (IF = 65.1), as shown in Table 3. One of the top 10 cited reference articles is Lao and Grady (2011) titled “Epigenetics and CRC”, a publication that discusses the relationship between epigenetics and CRC. In 2020, Jung et al. (2020) published the most cited article describing epigenetic modifications and regulators of CRC, which are important biomarkers for CRC.

**TABLE 3:** Top 10 highly cited references.

Item	Rank	Title	Journal	Citation
High-cited References	1	Epigenetics of colorectal cancer: biomarker and therapeutic potential	Nature Reviews Gastroenterology and Hepatology	36
	2	Epigenetics and colorectal cancer	Nature Reviews Gastroenterology and Hepatology	34
	3	Epigenetics in cancer	The New England Journal of Medicine	32
	4	Genome-scale analysis of aberrant DNA methylation in colorectal cancer	Genome Research	27
	5	Comprehensive molecular characterization of human colon and rectal cancer	Nature	27
	6	Epigenetic Alterations in Colorectal Cancer: Emerging Biomarkers	Gastroenterology	24
	7	Global Cancer Statistics 2020: GLOBOCAN Estimates of Incidence and Mortality Worldwide for 36 Cancers in 185 Countries	Ca-A Cancer Journal for Clinicians	21
	8	A decade of exploring the cancer epigenome - biological and translational implications	Nature Reviews Cancer	21
	9	Cancer epigenetics: from mechanism to therapy	Cell	19
	10	The consensus molecular subtypes of colorectal cancer	Nature Medicine	19

#### 3.3.3 Analysis of keyword co-occurrence, burst, and cluster

**FIGURE 12:**
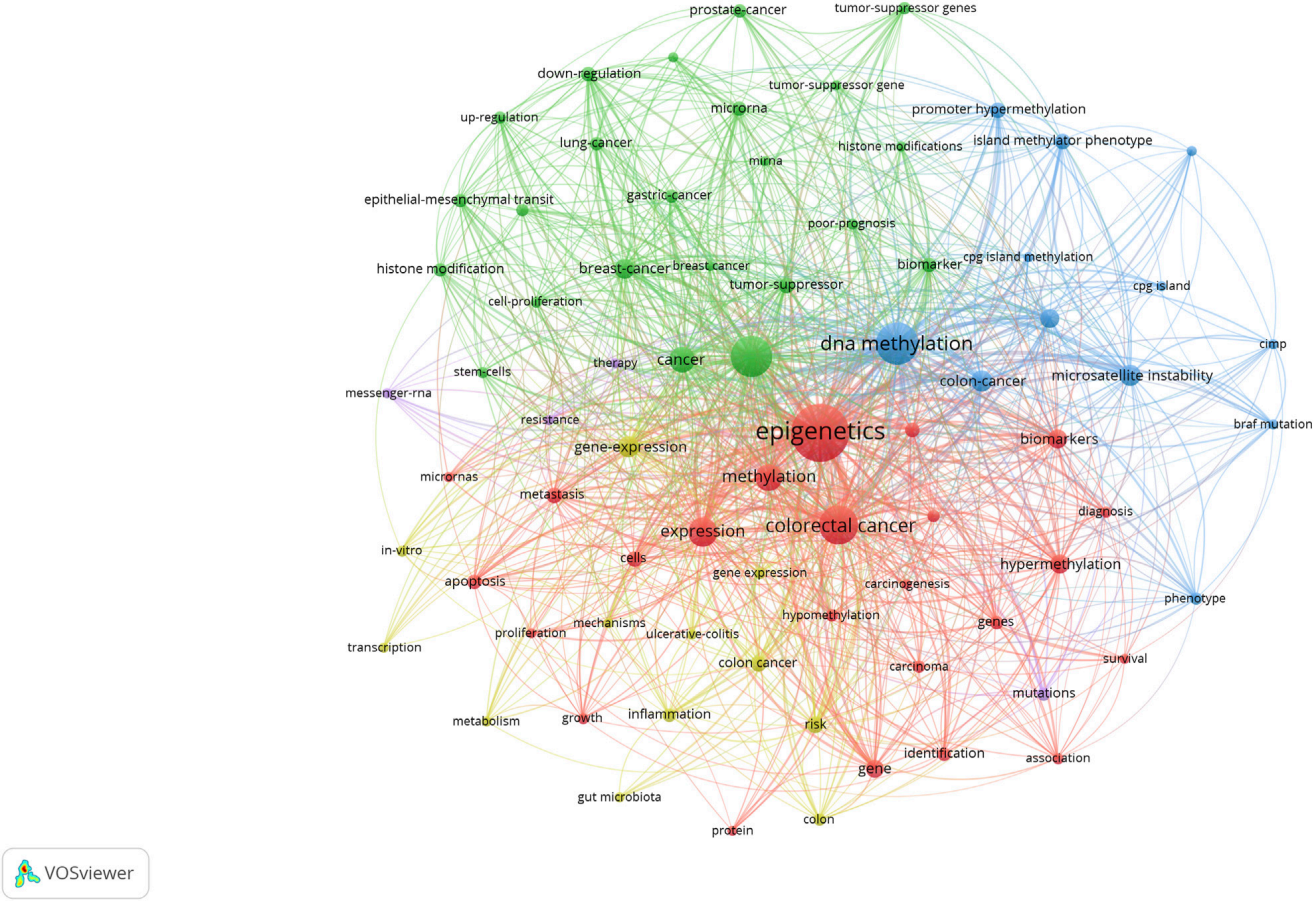
Keyword co-occurrence map of CRC and epigenetics.

High-frequency keywords indicate current research trends in this field. Table 3 shows the details of the keyword co-occurrence. The size of the nodes in the graph corresponds to the frequency of keywords. The keywords used to extract the keywords of the most common co-occurrence graph and the detailed information are as follows: CRC, DNA methylation, expression, epigenetics, colon cancer, gene expression, breast cancer, MSI, methylation, and promoter methylation (Figure 12). Table 3 shows the data of specific terms with a high ranking of keyword centrality: hypermethylation, tumor suppressor, gene expression, MSI, gastric cancer, cells, cell proliferation, expression, methylation, and gene. Through keyword co-occurrence and keyword centrality, we can see that current research focuses on the mechanism between CRC and epigenetics (including DNA methylation, gene expression, and MSI). From the keyword co-occurrence map of CRC epigenetic drugs, we found that 5-fluorouracil (5-FU), irinotecan, and oxaliplatin have received much attention from scientists (Supplementary Figure S2; Supplementary Table S1).

**FIGURE 13:**
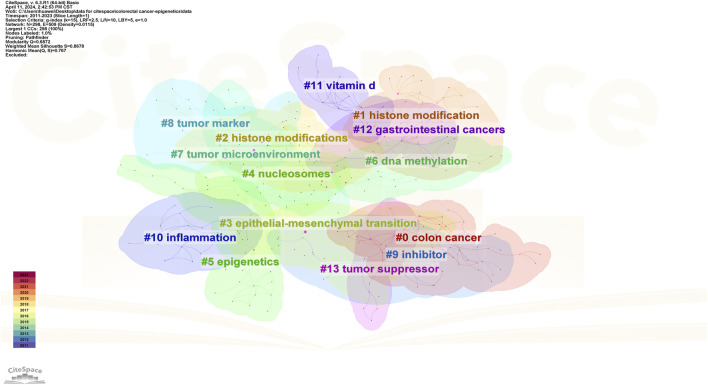
Keyword cluster map of CRC and epigenetics.

**FIGURE 14:**
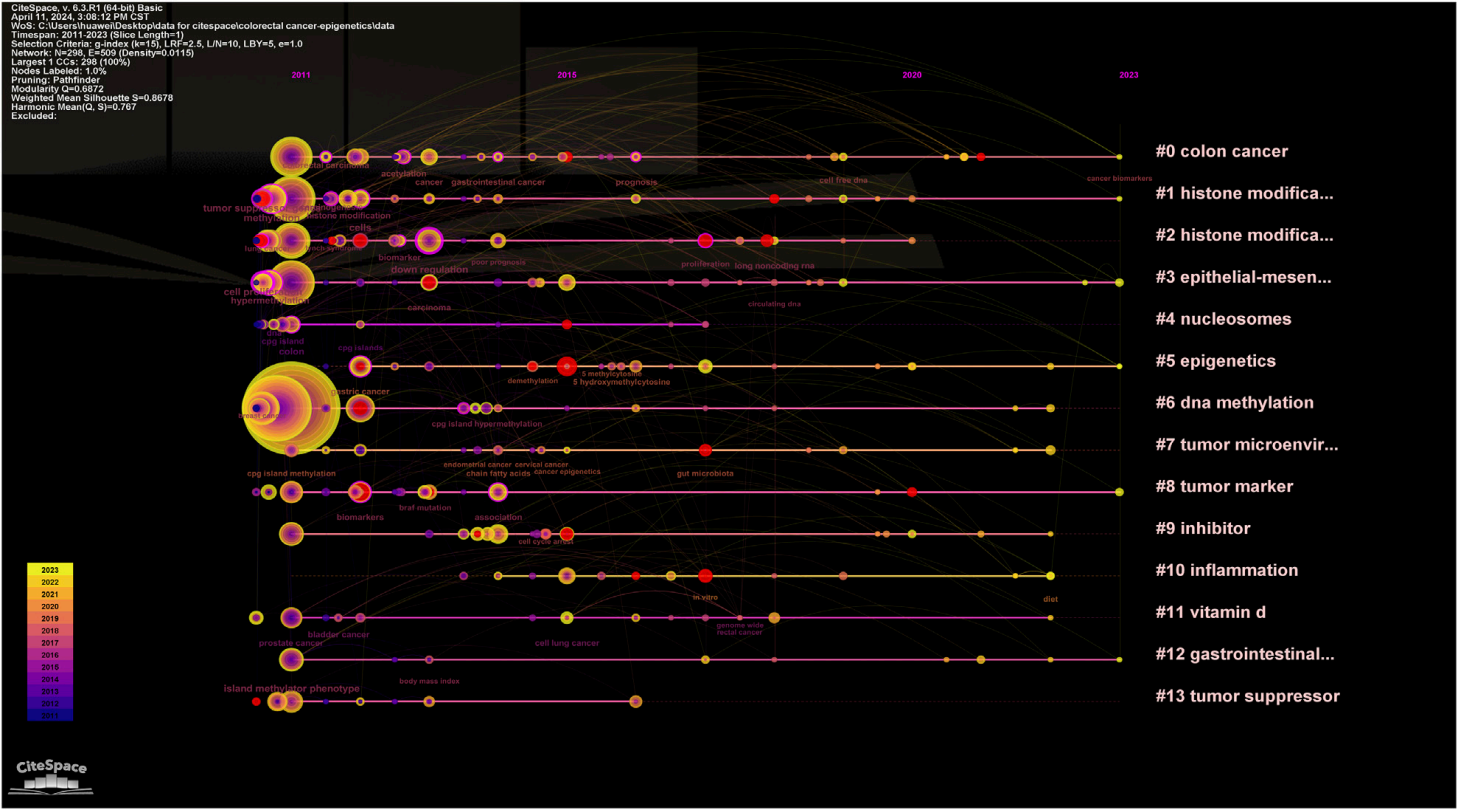
Keyword clustering timeline map of CRC and epigenetics.

In addition, CiteSpace uses an algorithm to cluster keywords close to the research field. The higher the cluster ranking, the more keywords contained in the cluster. Detailed information regarding keyword clustering is presented in Table 4; Figures 13. Figure 14 shows the timeline of the keyword clustering. A value greater than 0.6 in the silhouette table indicates the validity of the clustering. The study identified 14 clusters known as colon cancer, histone modification, histone modifications, epithelial and mesenchymal transition, nucleosomes, epigenetics, DNA methylation, tumor microenvironment, tumor markers, inhibitors, inflammation, vitamin D, gastrointestinal cancers, and tumor suppressors. Keyword clustering suggests that the mechanisms between CRC and epigenetics and how vitamin D mediates them are the focus of current research.

**TABLE 4:** Top 20 keywords in terms of frequency and centrality.

Rank	Keyword	Frequency	Keyword	Centrality
1	Colorectal cancer	592	Hypermethylation	0.13
2	DNA methylation	351	Tumor suppressor	0.11
3	Expression	167	Gene expression	0.09
4	Epigenetics	158	MSI	0.09
5	Colon cancer	134	Gastric cancer	0.09
6	Gene expression	108	Cells	0.09
7	Breast cancer	96	Cell proliferation	0.09
8	MSI	93	Expression	0.08
9	Methylation	73	Methylation	0.08
10	Promoter methylation	65	Gene	0.08
11	Gene	64	Island methylator phenotype	0.08
12	Hypermethylation	61	Breast cancer	0.07
13	Gastric cancer	50	Risk	0.07
14	Risk	49	Cancer	0.07
15	Island methylator Phenotype	48	Tumor suppressor genes	0.07
16	Tumor suppressor	48	CPG island methylation	0.07
17	Cells	45	Histone modifications	0.07
18	Promoter hypermethylation	45	Activation	0.07
19	Lung cancer	36	Cancer epigenetics	0.07
20	Downregulation	36	Biomarkers	0.06

**FIGURE 15:**
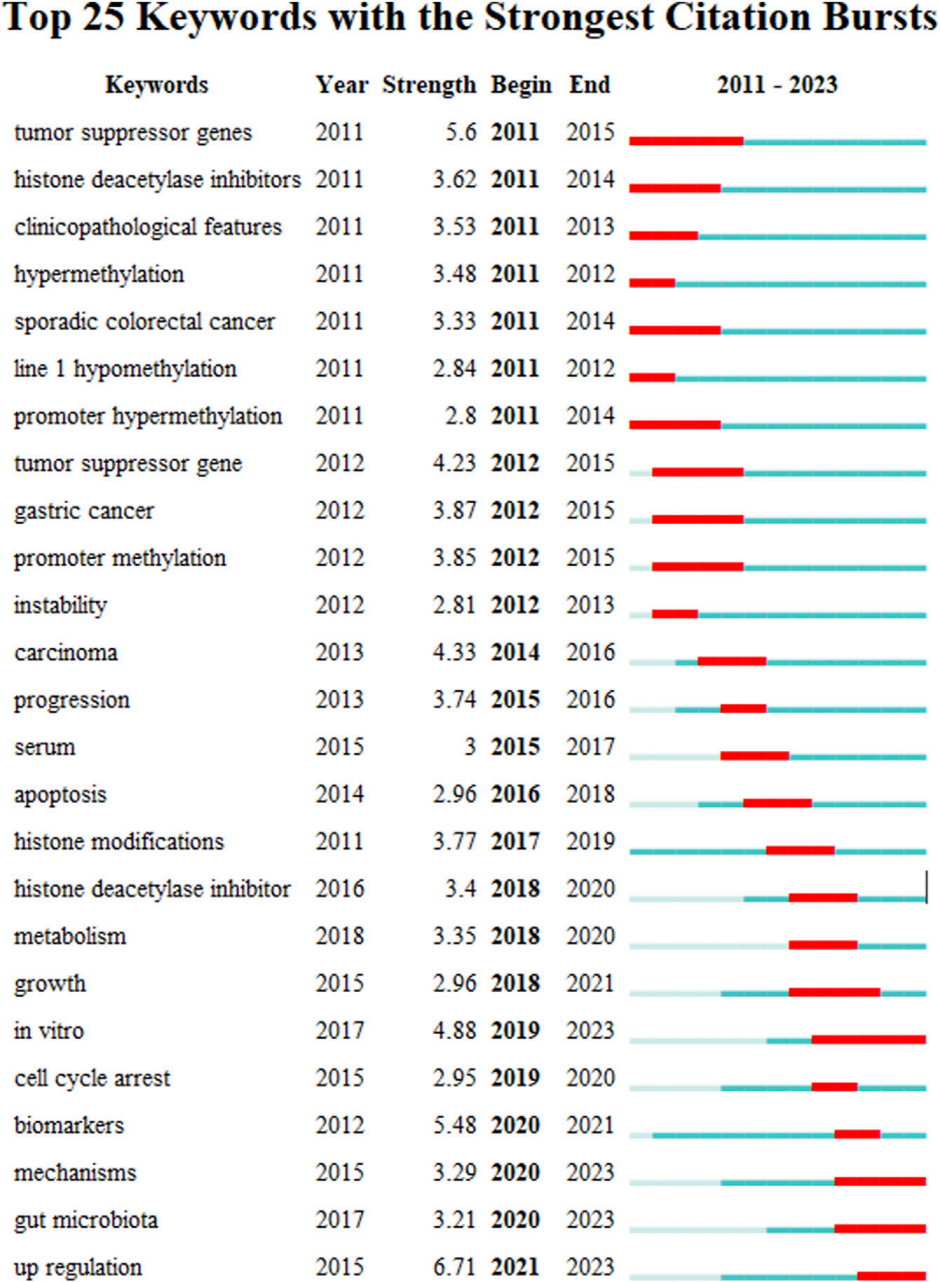
Keyword brusts map for CRC and epigenetics.

Keyword burst refers to a concentration of research content that appears over a period of time, indicating the future direction of research. Figure 15 and Table 5 show the top 25 keyword bursts in the study area, and the red line represents the duration of the keyword burst. In recent years, the keywords focus on “*in vitro*,” “mechanism,” “gut microbiota,” and “upregulation.” This means that how the gut microbiota mediates epigenetics and CRC may become a trend for future research in this field.

**TABLE 5:** Keyword cluster analysis.

Cluster	Size	Sihouette	Mean year	Label (LLR)	Other keywords
0	35	0.876	2015	Colon cancer	Beta catenin; tumor growth; *in vivo*; adenomatous polyposis coli
1	32	0.898	2014	Histone modification	Cells; dnmt3b; dna methylation; expression
2	30	0.745	2013	Histone modifications	Cancer progression; environmental exposures; therapeutic target; microrna
3	25	0.861	2016	Epithelial-mesenchymal transition	Epigenetics; breast cancer; hypermethylation; wnt/beta-catenin signaling pathway
4	25	0.795	2012	Nucleosomes	Cpg island; clinical outcome; Tumor suppressor genes; cytoskeleton
5	22	0.925	2017	Epigenetics	Dna methylation; immunotherapy; esophageal cancer; gastric cancer
6	20	0.79	2013	Dna methylation	dna methylation; epigenetics; colorectal cancer; breast cancer; promoter methylation
7	19	0.931	2016	Tumor microenvironment	Microbiome; gut microbiota; dysbiosis; resistance
8	19	0.93	2014	Tumor marker	Gene regulation; screening; mismatch repair; prognosis
9	17	0.984	2016	Inhibitor	Angiogenesis; dna methylation; gene; emt
10	17	0.891	2018	Inflammation	Inflammatory bowel disease; *in vitro*; nutrition; bioactive components
11	16	0.901	2015	Vitamin d	Cancer; ovarian cancer; bladder cancer; glycogene
12	11	0.842	2016	Gastrointestinal cancers	Liquid biopsy; extracellular vesicles; interdisciplinary; molecular epidemiology
13	10	0.839	2012	Tumor suppressor	Circulating tumor dna; pectin; n-3 polyunsaturated fatty acids; post-menopausal

## 4 Discussion

Epigenetics is crucial in CRC and is considered by researchers to be an important gene target for CRC (Dienstmann et al., 2017; Kawakami et al., 2015). This study analyzed publication trends, countries, institutions, authors, research priorities, and hotspots in order to improve our understanding of the role of epigenetics in CRC and promote innovative treatment strategies for CRC.

### 4.1 General information analysis

This study collected nearly 12 years of WoSCC data from this research field for relevant analysis. The cumulative number of publications has grown steadily over time, indicating an escalating interest of the scientific community in this area of research. The United States published the most papers (246 papers), followed by China and Italy. This shows that the United States has become a research powerhouse in this field because of its strong economic and policy support for related fields. As a developing country, China is prominent in the field of CRC and epigenetic research. This also shows the growing importance of cancer in developing countries. Harvard University, Harvard Medical School, and Harvard T.H. Chan School of Public Health are the top three institutions in this field, indicating that this research field is supported by the world’s most cutting-edge technology. Ogino et al., the most widely published author in the field, classified CRC into molecular categories, including KRAS, BRAF, MSI, and CIMP (Ogino et al., 2011).

Bibliometric analysis can assess collaboration between authors, institutions, and countries in a particular research area (Tian et al., 2023). Centrality represents the degree of cooperation among countries, institutions, and authors. The United States, Germany, United Kingdom, China, and Spain are the top five countries for centrality, representing the strongest collaboration in the field of research. Collaboration between institutions shows that the University of Texas System, Brigham and Women’s Hospital, Albert Einstein College of Medicine, Ruprecht Karls University Heidelberg, and Helmholtz Association have the closest cooperation and highest central position. Although Harvard University is the research institution with the largest number of publications in this field, it lacks cooperation with other institutions and should strengthen cooperative research in this area. Ogino, Shuji, Alwers, Elizabeth, Akimoto, Naohiko, Amitay, and Efrat L have connections with other researchers working together in this field. In addition, the centrality of the remaining authors is zero, which means that the institution and the state should develop corresponding policies to strengthen cooperation among authors. We believe that cooperation between relevant national institutions and personnel will contribute to the long-term development of this field of research.

### 4.2 Research focus and hotspot

The hot spots and frontiers of the research field are reflected in the bibliometrics. Based on the analysis of highly co-cited references, highly cited references, and keyword co-occurrence, the research focus of CRC and epigenetics is closely related to its mechanism. Therefore, we should focus on the CIMP and MSI. At the same time, we found that DNA methylation analysis of SEPT9 in plasma is helpful for the diagnosis and detection of CRC. Interestingly, in the keyword cluster analysis, we found that scholars were interested in vitamin D-mediated epigenetics and CRC. In addition, keyword burst analysis shows that scholars have paid increasing attention to how the gut microbiota mediates epigenetics and CRC in recent years, which may be the direction of future research in this field.

#### 4.2.1 Mechanisms of CRC and epigenetics—CIMP and MSI

CRC poses a serious threat to human health because of its high morbidity and mortality (Wei et al., 2020). Accumulation of epigenetic changes leads to carcinogenesis of the normal glandular epithelium, which leads to the occurrence and development of CRC (Fearon and Vogelstein, 1990). Epigenetic changes can inactivate DNA repair and cancer suppressor genes (Bonasio et al., 2010). An increasing number of studies have shown that epigenetic changes, such as DNA methylation, histone modification, nucleosome localization, and non-coding RNA, play key roles in the occurrence and development of CRC (Okugawa et al., 2015). In recent years, research on DNA methylation modification has received extensive attention. DNA methylation occurs at the fifth carbon position of CpG dinucleotides of cytosine residues. Approximately 60%–80% of CpG cytosine methylation occurs in human cells. DNA methylation rich in cytosine bases in cg sequences, called CpG islands, is primarily located near the transcriptional start sites of compositional unmethylated promoter genes (Goel and Boland, 2012). Toyota et al. first proposed a new CIMP-positive subgroup of CRC in 1999 that showed a wide range of DNA hypermethylation in CRC tissues (Toyota et al., 1999). CIMP is now recognized as the initial event in the development of CRC-serrated tumors and is a distinct molecular subtype of sporadic CRC (Advani et al., 2019). The CIMP subtype is characterized by a high frequency of methylation of genes (Toyota and Issa, 1999). CIMP promotes hypermethylation of tumor suppressor genes through DNA methyltransferase (DNMT), leading to transcriptional inactivation of tumor suppressor genes and the chronic development of CRC (Miranda Furtado et al., 2019). A meta-analysis showed that CIMP was significantly associated with the prognosis of CRC (Juo et al., 2014). In the past 20 years, CIMP has been considered a popular research area for CRC.

The Cancer Genome Atlas (TCGA) research network conducted an in-depth analysis of 224 pairs of CRC and normal tumor genomes and found that 77% of CRCS tumors had high-frequency MSI (MSI-H) (Cancer Genome Atlas Network, 2012). DNA mismatch repair (dMMR) defects are present in approximately 12%–15% of CRC and manifest as MSI. dMMR/MSI CRC develops from germline mutations in MMR genes (MLH1, MSH2, MSH6, PMS2) and has unique features, including a preference for the proximal colon, poor differentiation, and abundance of tumor-infiltrating lymphocytes (Kawakami et al., 2015). Microsatellites consist of single nucleotide, dinucleotide, or high-order nucleotide repeat sequences. These gene sequences are most susceptible to mutations that lead to the development of MSI (Jiricny, 2006). The MSI phenotype mediates the mutation of CRC genes, particularly BRAF and MRE11A, as well as other genes such as KRAS, of which clinical researchers are increasingly interested in the genetic mutation of CRC, largely because of its important role in the development of tumors and its potential therapeutic targets and value (Vogelstein et al., 1989). In MMR-defective CRC, multiple genes are mutated in MSI that are associated with cell functions and pathways, such as DNA repair proteins, growth factors, pro-apoptotic factors, mismatch repair proteins, and histone-modifying factors (Duval and Hamelin, 2002). Therefore, these genes and pathways could serve as potential drug targets and biomarkers. In terms of the prognosis of CRC, several studies and meta-analyses have confirmed that MSI tumors are not prone to spread and metastasis, and the prognosis is good (Gryfe et al., 2000). Therefore, there may be a clinical need to consider incorporating MSI testing into routine CRC testing to inform patient prognosis and guide treatment decisions.

The discovery of epigenetic mechanisms of MSI and CIMP has led to new therapeutic targets and drugs for CRC. 5-FU, irinotecan, and oxaliplatin are representative epigenetic drugs. Adjuvant chemotherapy with 5-FU can provide survival benefits in CRC patients with CIMP positive status (Van Rijnsoever et al., 2003). 5-FU disrupts DNA replication mainly by inhibiting thymidylate synthase (Weng and Huang, 2024). However, a study have shown that 5-FU has the characteristics of low treatment rate, large individual differences and susceptibility to drug resistance, and the obvious individual epigenetic differences may be one of the reasons (Vodenkova et al., 2020). Irinotecan appears to be a potential biomarker for CRC chemotherapy in CIMP positive status. Irinotecan activates multiple cancer cell signaling pathways through demethylation, increasing efficacy against CRC and reducing toxicity in humans (Tsai et al., 2012; Sharma et al., 2017). The main side effects of irinotecan in CRC patients include: bradycardia, sweating, tearing, abdominal pain, and diarrhea (divided into early-onset and late-onset diarrhea) (Tsuboya et al., 2019). Oxaliplatin has been shown to be associated with the expression of MSI-enriched genes in CRC (Condelli et al., 2021). Oxaliplatin achieves anti-tumor effects by forming DNA adducts (Kweekel et al., 2005). Peripheral neurotoxin is the main adverse reaction of oxaliplatin, acute peripheral neurotoxin symptoms are cold sensitivity and limb neuropathic pain, autonomic dysfunction can be complicated by chronic peripheral neurotoxin (Kang et al., 2021).

#### 4.2.2 SEPT9 for diagnosis and detection of CRC

One of the reasons for the higher incidence and mortality of CRC is the low rate of early detection. Although colonoscopy can increase the probability of early detection of CRC, colonoscopy is an invasive procedure and may affect patients’ willingness to be screened for CRC early (Yörüker et al., 2016). Epigenetics can regulate CRC gene expression through abnormal methylation, which is one of the most effective methods for early detection of CRC-related cancer markers (Toyooka et al., 2002; Toyota et al., 2000). The study found that methylated DNA concentrations were significantly elevated in the blood of cancer patients; therefore, the development of CRC-related blood tests could increase screening in the early stages of CRC (Herrera et al., 2005; Sabbioni et al., 2003). Methylated SEPT9 DNA (mSEPT9) is an assay that compares methylation markers in normal colon and CRC tissues (Lofton-Day et al., 2008). More than 90% of tumor tissues have a higher relative amount of mSEPT9 than normal colon mucosal tissue, and studies have shown that positive plasma mSEPT9 may indicate the occurrence of CRC, with a sensitivity between 52% and 72% and specificity between 90% and 95% (Grützmann et al., 2008; deVos et al., 2009). However, the effectiveness and cost of screening CRC using mSEPT9 still need to be further evaluated (Church et al., 2014).

#### 4.2.3 Vitamin D mediates epigenetics and CRC

Vitamin D mediates CRC development of CRC through genetic and epigenetic effects (Khayami et al., 2022). It has been reported that cumulative methylation levels of genes associated with the vitamin D metabolic pathway may contribute to CRC risk (Wang et al., 2023). CpG islands are present in all genes associated with vitamin D metabolic pathways that undergo gene silencing via hypermethylation (Fetahu et al., 2014). Studies have also shown that the vitamin D active substance 1α,25-dihydroxyvitamin D+3 (1,25(OH)2D3, calcitriol) can promote SIRT1 activation in colon cancer cells, and SIRT1 activators may provide new therapeutic possibilities for patients with VD deficiency or non-response to colon cancer (Carding et al., 2015). Vitamin D is still the forefront and hotspot of current research in this field and deserves the attention of researchers.

#### 4.2.4 Gut microbiota mediates epigenetics and CRC

The interactions between the gut flora and the host regulate various physiological processes, such as digestion and absorption of food, synthesis of vitamins and bile acids, development of epithelial and mucosal layers, regulation of innate and mucosal immunity, and disruption of the balance of beneficial gut microbes, which can lead to the development of chronic inflammation, ultimately leading to the development of CRC (Hu et al., 2015). Epigenetics mediate this process. A variety of miRNAs associated with CRC progression are significantly correlated with gene expression (Gao et al., 2009). Metabolites produced by the gut flora, such as butyrate, regulate the expression of various miRNAs in CRC (Jones, 2012). DNA methylation is an epigenetic modification in which the donor metabolite s-adenosylmethionine (SAM) plays an important role (Wasson et al., 2006). Gut microbes are the main producers of folic acid, which is involved in SAM synthesis. Folate deficiency leads to DNA hypomethylation, which mediates the emergence of CRC (Chen et al., 2022). Patients with CRC receiving chemotherapy show altered gut microbiome composition, and one of the most common side effects of chemotherapy drugs such as irinotecan and 5-FU is diarrhea (Sanders et al., 2019). Treatment-induced diarrhea is mainly treated by improving the gut microbiota, administering prebiotics, probiotics, and fecal transplantation (FMT) (Ting et al., 2022).

#### 4.2.5 Epigenetic drug therapy and clinical applications for CRC

Evidence for epigenetics at every stage of colorectal cancer progression is growing, and patients with colorectal cancer may benefit from epigenetic therapy. The DNMTi: 5- azacytidine (azacitidine or 5-azaCR or Vidaza) and its deoxy derivative 5-aza-2- deoxycytidine (5-azaCdR or decitabine) are the most studied DNA methylation inhibitor. These drugs form irreversible covalent bonds at targeted methylation sites that impede the occurrence of DNA methylation and thus hinder the progression of CRC (Puccini et al., 2017). HDACi targets histones through the accumulation of acetylated histones which ultimately leads to cell arrest and apoptosis. The potential activity of EGFR/HER2 inhibitor (lapatinib) combined with HDACi Panobinostat in colon cancer cells has been demonstrated, and further evaluation of the efficacy of this combination in the treatment of CRC is warranted (LaBonte et al., 2011). Decitabine in combination with panizumab (monoclonal antibody -mAb against EGFR) has been shown to provide partial remission in patients with wild-type KRAS mCRC (Garrido-Laguna et al., 2013). Vitamin C can selectively kill CRC cells with KRAS and BRAF mutations (Yun et al., 2015). In addition, vitamin C and 5-azacitidine can synergically inhibit CRC cancer cells (Liu et al., 2016). Because of its low cost and toxicity, vitamin C combined with DNMTi may be a good treatment option for CRC patients with KRAS and BRAF mutations. There are a number of Phase I/II clinical trials investigating the efficacy of epigenetic agents for CRC, suggesting that epigenetic therapy may be a new hope for future colorectal cancer patients.

The early screening of CRC is critical, and colorectal scopy is the gold standard for CRC diagnosis. However, due to the invasive nature of colorectal cancer and the high cost of treatment, and the possible side effects such as bleeding, epigenetic factors including DNA methylation, such as VIM gene methylation, SFRP2 methylation, etc., have broad prospects for the future diagnosis and prediction of CRC (Jung et al., 2020).

## 5 Limitations of the research

Our study has some limitations. The WOSCC database served as our research database, and limiting our results to the English language may have resulted in missing literature. Second, changes in the format of the names of certain authors or institutions in the WOSCC may lead to bias in the statistical analysis. Finally, this study cannot guarantee that every publication fully meets the search criteria. However, we provided sufficient results and analyses to reflect the current state of the research field.

## 6 Conclusion

This study quantified 12 years of research on CRC and epigenetics using bibliometrics and visual analysis of the WOSCC database. The countries, institutions, and authors with the largest number of published articles were the United States, Harvard University, and Ogino and Shuji, respectively. In summary, the outstanding research areas in CRC and epigenetics are as follows: SEPT9 is a blood test for the early detection of CRC. Vitamin D and gut microbiota mediates CRC and epigenetics, and probiotics may alleviate CRC-related symptoms. CIMP and MSI are important epigenetic mechanisms of colorectal cancer. 5-FU, irinotecan, and oxaliplatin are currently the main representative drugs, but a large number of high-quality clinical trials are still needed to confirm their efficacy and safety. More epigenetic mechanisms related to CRC progression need to be discovered and studied. Current studies have found that epigenetic therapy such as 5-Aza-CdR/SGI-110 and vitamin C can inhibit DNA methylation of CRC. The development and targeted transportation of DNA methylation inhibitors, as well as the combined use of DNA methylation inhibitors with targeted drugs and cytotoxic drugs may be the future research direction, which is the good news for CRC patients. Interdisciplinary research into epigenetics, pharmacology and clinical research is recommended to develop more effective treatments for CRC. This study reviewed the current research trends and hotspots between CRC and epigenetics timely, which has important implications for the field.

## Data Availability

The original contributions presented in the study are included in the article/Supplementary Material, further inquiries can be directed to the corresponding author.

## Supplementary material

SUPPLEMENTARY FIGURE S1 Annual publication of epigenetic drugs in colorectal cancer.

SUPPLEMENTARY FIGURE S2 Keyword co-occurrence map of epigenetic drugs for colorectal cancer.
