# Supplementary figures and images for: The mechanisms and drug therapies of colorectal cancer and epigenetics: bibliometrics and visualized analysis
Source: Front Pharmacol. 2024 Aug 29;15:1466156. doi: 10.3389/fphar.2024.1466156 (PMC11391208; doi:10.3389/fphar.2024.1466156)

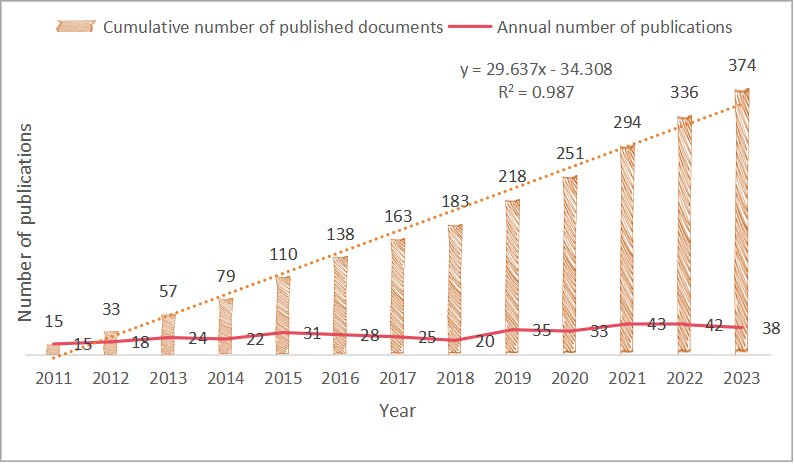

Supplement: Supplementary file 1 [file Image1.TIF]

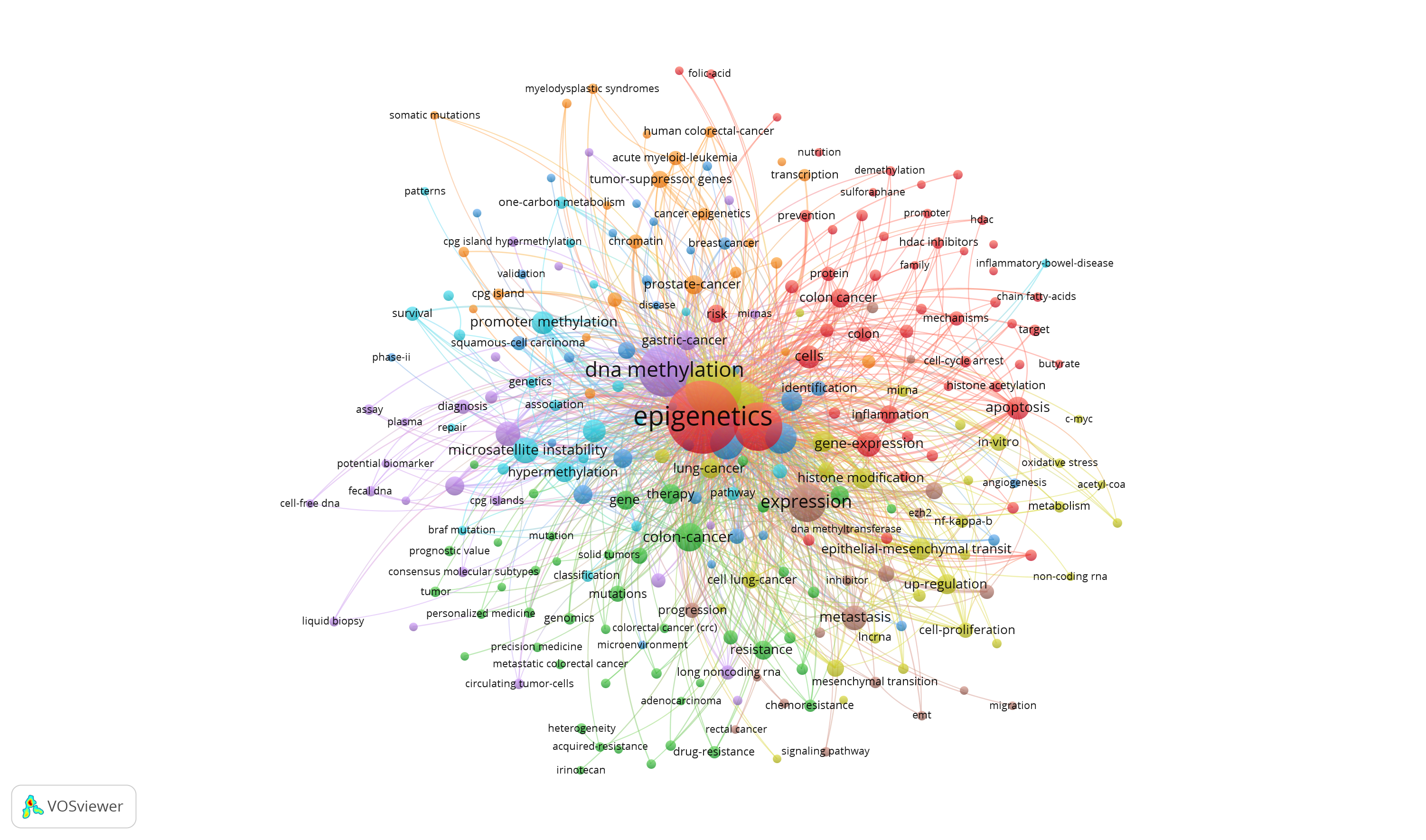

Supplement: Supplementary file 3 [file Image2.TIFF]
